# Role of flavonoids in inhibiting triple-negative breast cancer

**DOI:** 10.3389/fphar.2024.1411059

**Published:** 2024-08-27

**Authors:** Shuai Wang, Kuanyu Wang, Cheng Li, Jing Chen, Xiangding Kong

**Affiliations:** ^1^ Graduate School, Heilongjiang University of Chinese Medicine, Harbin, China; ^2^ The Second Department of Surgery, The First Affiliated Hospital of Heilongjiang University of Traditional Chinese Medicine, Harbin, China

**Keywords:** triple-negative breast cancer, flavonoids, traditional Chinese medicine, breast cancer, pharmacology

## Abstract

Increasing incidences of metastasis or recurrence (or both) in triple-negative breast cancer (TNBC) are a growing concern worldwide, as these events are intricately linked to higher mortality rates in patients with advanced breast cancer. Flavonoids possess several pharmaceutical advantages with multi-level, multi-target, and coordinated intervention abilities for treating TNBC, making them viable for preventing tumor growth and TNBC metastasis. This review focused on the primary mechanisms by which flavonoids from traditional Chinese medicine extracts inhibit TNBC, including apoptosis, blocking of cell cycle and movement, regulation of extracellular matrix degradation, promotion of anti-angiogenesis, inhibition of aerobic glycolysis, and improvement in tumor microenvironment. This review aims to improve the knowledge of flavonoids as a promising pharmacological intervention for patients with TNBC.

## 1 Introduction

Approximately 13% of women globally are diagnosed with breast cancer (Giaquinto et al., 2022). Triple-negative breast cancer (TNBC) is a kind of cancer where the estrogen receptor (ER), progesterone receptor, and human epidermal growth factor receptor 2 (HER-2) are not expressed. TNBC exhibits a low differentiation rate, high invasiveness, and high metastasis and recurrence (Lu et al., 2023). This type of breast cancer makes up 12% of all cases in the US and has a 5-year survival rate that varies from 8% to 16% (Howard and Olopade, 2021). The treatment options for TNBC include surgical intervention, radiation therapy, chemotherapy, targeted therapy, and immunotherapy. However, limited therapeutic strategies for TNBC due to a lack of effective biological targets and biomarkers, diverse molecular subtypes, and complex biological behaviors and clinical characteristics have made it a significant clinical challenge for years. Therefore, developing more efficient therapeutic drugs is necessary.

Flavonoids derived from TCM exhibit a diverse array of anti-tumor properties that can be combined with modern therapies to improve treatment efficacy and prevent the occurrence of breast cancer. Flavonoids extracted from TCM have wide-ranging effects linked to multiple cancer-related signaling pathways. This review briefly introduces flavonoids from TCM extracts, their targets, and potential mechanisms for TNBC.

## 2 Molecular pathogenesis of breast cancer

### 2.1 Breast cancer cell apoptosis

A disequilibrium between cellular division and cell death leads to uncontrolled proliferation of cancer cells. Apoptotic signaling pathways can be categorized into three distinct types: intrinsic, extrinsic, and endoplasmic reticulum pathways. Apoptosis evasion mechanisms can be roughly classified into three types: diminished caspase function, impaired death receptor signaling, and an altered balance of pro-apoptotic and anti-apoptotic proteins. This is related to the disruptions in the equilibrium of the Bcl-2 family, defects in the p53 tumor suppressor gene, anomalous expression of apoptosis protein inhibitors, decreased caspase activity, and impaired death receptor signaling (Singh and Lim, 2022).

### 2.2 Cell cycle arrest

Cell cycle dysregulation is the basis of uncontrolled cell proliferation. Cells that have lost the checkpoint mechanisms have highly unstable genomes. In breast cancer, cell cycle abnormalities are frequently observed, including the loss of Rb function, inhibitors, and increased abundance of cyclin D, cyclin E, and cyclin-dependent kinase (CDK). Cyclin D1 controls how cells move from the G1 phase, where they prepare for DNA replication, to the phase where they actually copy their DNA. This gene is also important for preventing human breast cancer cells from growing too much. There is a strong correlation between the elevated expression of CCND1, which codes for the protein Cyclin D1, and decreased survival rates (Aftab et al., 2021). Cyclin E serves as a potent prognostic indicator of breast cancer and plays a crucial role in determining tumor aggressiveness and predicting the recurrence of TNBC (Guerrero Llobet et al., 2020).

### 2.3 Regulation of extracellular matrix degradation

Breast cancer cells become potentially malignant after penetrating and dissolving the extracellular matrix (ECM), particularly the basement membrane. Epigenetic mechanisms are important for regulating EMT/ECM-related pathways. When cancer cells acquire mesenchymal features, it promotes the advancement of TNBC, and the process of DNA methylation and the action of enzymes that modify histones contribute to changes in ECM/EMT alterations in TNBC (Zolota et al., 2021). Matrix metalloproteinases (MMPs) are proteolytic enzymes containing active Zn^2+^. MMP9 degrades the ECM near tumors, which are intimately associated with tumor invasion and metastasis, performs the final degradation of collagen fibers, and removes malignant cells from the complicated network around them. Collagen is a significant metabolite of connective tissue that undergoes a two-step degradation process in mammary gland tissue. MMP-2 and MMP-9 specifically target the breakdown of denatured interstitial collagen or gelatin, as well as collagen types IV and V found in the basement membrane. MMP-1 is the sole MMP enzyme that can degrade all types of collagen in the mammary gland and is crucial in breaking down stromal fibers in various diseases (Argote Camacho et al., 2021). MMP9 is crucial for the development of the “metastatic niche” and controls the spread of cancer cells to the lungs when overexpressed. The coexpression of MMP-1, MMP-2, and MMP-9 may indicate an unfavorable prognosis in patients with breast cancer (Mohammadian et al., 2020; Jiang and Li, 2021).

### 2.4 Inhibition of the epithelial-mesenchymal transition (EMT)

Epithelial–mesenchymal transition (EMT) is a significant process that triggers tumor invasion and metastasis. Cancer metastasis can be divided into multiple phases, including *in situ* tumor growth, EMT, invasion, infiltration, survival in blood circulation, extravasation, dormancy, and metastatic growth (Fares et al., 2020). EMT involves the disruption of cell adhesions in cancer cells originating from epithelial tissue and the upregulation of particular metabolites in the constricted cytoskeleton, leading to a mesenchymal phenotype characterized by increased invasiveness and the migration of primary cancers. TNBC cells evolve from epithelial cells to hybrid epithelial/mesenchymal (E/M) and strong mesenchymal patterns during invasion. Similarly, during colony formation, TNBC cells transition from epithelial cells to a hybrid E/M state (Grasset et al., 2022). EMT is commonly identified by loss of cytokeratin and E-cadherin and gain of mesenchymal-associated molecules, N-cadherin, fibronectin, and vimentin. EMT involves many signaling pathways, including NF-κB, TGF-β, Akt, Wnt, Notch, PPARγ, and RAS, and is also affected by hypoxia and microRNA (miRNA) expression. The transcription factors TWIST, Snail, and Zeb1/Zeb2, as well as epigenetic regulators, miRNAs, and alternative splicing, are regulated by these signaling pathways during breast cancer growth.

### 2.5 Breast cancer stem cells

Breast cancer stem cells (BCSCs) are stem cells within a tumor that possess the ability to regenerate themselves and have the potential to develop into malignancies. The variation in tumors among different individuals can be ascribed to the inherent molecular subtypes of breast cancer, while the variation within a tumor can be elucidated by the concept of cancer stem cells, which primarily infiltrate the adjacent mesenchyme or enter the circulatory system through EMT during tumor metastasis. BCSCs undergo a mesenchymal-epithelial transition (MET) to form massive metastatic colonies in distant organs (Lu and Kang, 2019). BCSCs show higher metastatic potential due to the upregulation of proteins associated with cell metastasis and motility, as well as the downregulation of adhesion proteins. Tumorigenicity is demonstrated by the activation of many pathways associated with BCSCs. Furthermore, The ability of BCSCs to easily transition between EMT and MET is essential for promoting both EMT and metastasis in breast cancer. In addition, immunosuppressive cells are recruited by BCSCs to promote breast cancer progression. BCSCs maintain quiescent to prevent elimination by immune cells that are effective in their function, or they can establish a microenvironment that suppresses the immune system by attracting populations that inhibit immune detection (Tallerico et al., 2017). BCSCs of NTBC can be identified using specific markers, such as CD44, CD24, CD133, and aldehyde dehydrogenase (Brugnoli et al., 2019). They are also influenced by various signaling pathways, such as BMP2, Wnt, NF-κB, Notch, STAT3, and Hedgehog, which regulate their growth, survival, and migration.

### 2.6 Anti-angiogenesis

Angiogenesis provides nourishment and oxygen to tumor tissue and disseminates cancer cells via blood vessels. Tumor blood vessels exhibit structural instability and dysfunction, leading to inflammation and tissue fibrosis, DNA hypermethylation, genomic instability, transdifferentiation, immunosuppression, growth, invasion of tumor cells, and resistance to apoptosis (Martin et al., 2019). Stagnation of blood flow results in reduced vascular permeability and blood concentration, thereby reducing the pH in tissues and inducing hypoxia. Activated hypoxia-inducible transcription factors (HIFs) stimulate angiogenesis, leading to higher invasiveness and/or resistance to treatment (Kao et al., 2023). Furthermore, due to the widespread occurrence of vascular leakage in tumors, tumor cells invade the systemic circulation, leading to metastasis (Tomita et al., 2021). The dysregulation of tumor-associated angiogenesis is controlled by diverse molecular elements, including vascular endothelial growth factor (VEGF), TGF-β-1, Interleukin (IL)-8, CD34, CD31, Factor VIII, angiopoietin-1, angiopoietin-2, platelet-derived growth factor, and fibroblast growth factor (FGF)-2.

### 2.7 Suppression of aerobic glycolysis

Abnormal metabolism is one of the most significant characteristics of malignancy. The Warburg effect, a feature of cancer cell energy metabolism, refers to the ability of tumor cells to utilize glycolytic products to synthesize their growth requirements under normoxic or hypoxic conditions. Despite the presence of sufficient oxygen, most tumor cells, including those in breast cancer, produce a substantial amount of energy through hyperglycolytic metabolism. Aerobic glycolysis closely regulates the proliferation and survival of breast cancer cells. Elevated levels of aerobic glycolysis hinder the effectiveness of cancer treatment and promote resistance to therapeutic agents. Aerobic glycolysis is assessed using fluorodeoxyglucose positron emission tomography (FDG-PET) and is used to monitor cancer recurrence and metastasis. The aerobic glycolytic pathway involves several key enzymes, such as hexokinase (HK), phosphofructokinase (PFK), pyruvate kinase, and glucose transporters. TNBC has distinct hypoxic characteristics and demonstrates abundant expression of HIF-1α (Liu et al., 2022). Other signaling pathways such as PI3K/Akt, mammalian target of rapamycin (mTOR), and AMP-activated protein kinase, along with transcription factors such as c-Myc, p53, and HIF-1 are also overexpressed in TNBC.

### 2.8 Improvement in tumor microenvironment

#### 2.8.1 Tumor-associated macrophages

Tumor-associated macrophages (TAMs) are the primary immune cells in the microenvironment of breast tumors. These macrophages enhance the growth of breast tumors by inducing breast cancer stemness, controlling energy metabolism, stimulating angiogenesis, drug resistance and cancer cell metastasis, and supporting immune system suppression (Munir et al., 2021). These mechanisms include the secretion of inhibitory cytokines, promotion of regulatory T cells (Tregs), and reduction of effector functions of tumor-infiltrating lymphocytes. TAMs regulate PD-1/PD-L1 expression. TAMs demonstrate a significant level of cellular plasticity, and changes in the TME lead to the transformation of TAMs into M1 macrophages, which mediate anti-tumor immune responses (Huang et al., 2022).TNBC induces M2 macrophage polarization, which positively feedback promotes the malignant evolution of TNBC cells. M2 promotes the migration of TNBC cells and induces angiogenesis in TNBC. Reversing M2 polarization is considered a target for cancer treatment (Meng et al., 2022).

#### 2.8.2 Chemokines

The TME comprises a heterogeneous combination of immune cells and soluble mediators, including chemokines, cytokines, and growth factors. These metabolites are found within or close to tumors. Chemokines function as immunological mediators, attracting and recruiting particular subsets of these cells into the TME and promoting tumor growth or regression. Some chemokines, such as CXCL9, CXCL10, and CCL16, can inhibit the growth of breast cancer cells. Other chemokines, such as CCL2, CCL5, CXCL8, and CXCL12, promote the growth of breast cancer (Masih et al., 2022).

#### 2.8.3 Bone microenvironment

In the bone microenvironment, diverse cell types, including osteocytes, adipocytes, endothelial cells, and neural cells, are essential in maintaining bone homeostasis. The development of bone metastases is a selective and multistep process. The growth, dormancy, and metastasis in the bone microenvironment linked to breast cancer are influenced by various bone marrow environments, including the endosteal (comprising osteoblasts, osteoclasts, and adipocytes) and vascular niches. After tumor cells invade the bone, they rely on stromal cells to further their survival and proliferation. Therefore, the intricate equilibrium between bone resorption and generation is disturbed. Osteolytic lesions constitute most breast cancer metastases. Bone metastases from breast cancer are characterized by osteoclastic bone resorption. Osteoclasts modulate bone resorption and promote the activation of malignancy cells (Zarrer et al., 2020).

#### 2.8.4 miRNAs

MicroRNAs (miRNAs) are a group of small molecule RNAs that are evolutionarily conserved and do not encode proteins. They are usually 21–23 nucleotides long and regulate the translational level of gene expression. miRNAs are abnormally expressed in various tumor types and are linked to several biological functions, including immunoregulation and cell metabolism. Therefore, they are used for cancer diagnosis, treatment, and prognosis. The primary oncogenic miRNAs in TNBC include miR-25-3p, miR-93, miR-21, and miR-455-3p. The primary miRNAs that suppress tumor growth in breast cancer are miR-29c, miR-30a-5p, miR-34a, miR-101, miR-130a, miR-134, miR-200a/b/c, miR-203, miR-206, miR-211, miR-223, miR-269-5p, miR-342-5p, miR-316-5p, miR-378, miR-384, miR-190-3p, miR-603, miR-613, miR-1296, and miR-4306 (Xu et al., 2020).

### 2.9 Hormones

Phytoestrogen are subtype of flavonoids and can mimic or induce estrogen-like responses. Their hydroxyl groups and phenolic rings, which are necessary for binding ERα and ERβ, make them similar to the most significant form of E2. Thus, ER, ERα, and ERβ can interact with the bioavailable phytoestrogens.In breast tissue, ERα activation promotes cell proliferation while ERβ decreases it and promotes apoptosis. In TNBC, the effects triggered by E2 are not only conducive to tumor growth but can also have anti-tumor properties, such as through the activation of ERβ. Decreased expression of mitochondrial ERβ led to a decline in mitochondrial activity in TNBC cells, promoting their growth through glycolysis and contributing to tumor advancement. Conversely, the enforced overexpression of mitochondrial ERβ reduced the proliferation of TNBC cells (Song et al., 2019). ERβ suppresses the growth of TNBC cell lines and also hinders their involvement in the formation of new blood vessels, invasion, and spread to other parts of the body. The ER-Beta agonist S-equol can effectively suppress the growth of TNBC by reducing the levels of Ki-67 (Lathrop et al., 2020).Chronic stress can contribute to the development of cancer. One of the most significant mechanisms is the continuous release of neurotransmitters caused by chronic stress, which ultimately leads to the activation of β2-adrenergic receptors (β2-AR) (Bernabé, 2021).

### 2.10 Drug resistance

Standard chemotherapy remains the cornerstone of systemic therapy, but TNBC often becomes resistant to cytotoxic drugs (Ou et al., 2024). According to the National Comprehensive Cancer Network guidelines, anthracyclines, taxanes, anti-metabolites, and microtubule inhibitors are preferred for chemotherapy. TNBC drug resistance mechanisms include antioxidant activity, mediation of drug efflux, reduction of intracellular drug accumulation, mediation of intracellular detoxification of cytotoxic drugs, enhanced DNA repair, anti-apoptosis, anti-autophagy, metabolic reprogramming, EMT, TME heterogeneity, and immune evasion (Bai et al., 2021). One potential approach to enhance the effectiveness of therapies and minimize their adverse effects involves reversing medication resistance and enhancing sensitivity to chemotherapy. Therefore, it is crucial to choose medications that are less toxic and more effective for patients with cancer. Therefore, flavonoids have been investigated as potent chemosensitizers in conjunction with standard chemotherapeutic drugs.

## 3 Flavonoid

**FIGURE 1:**
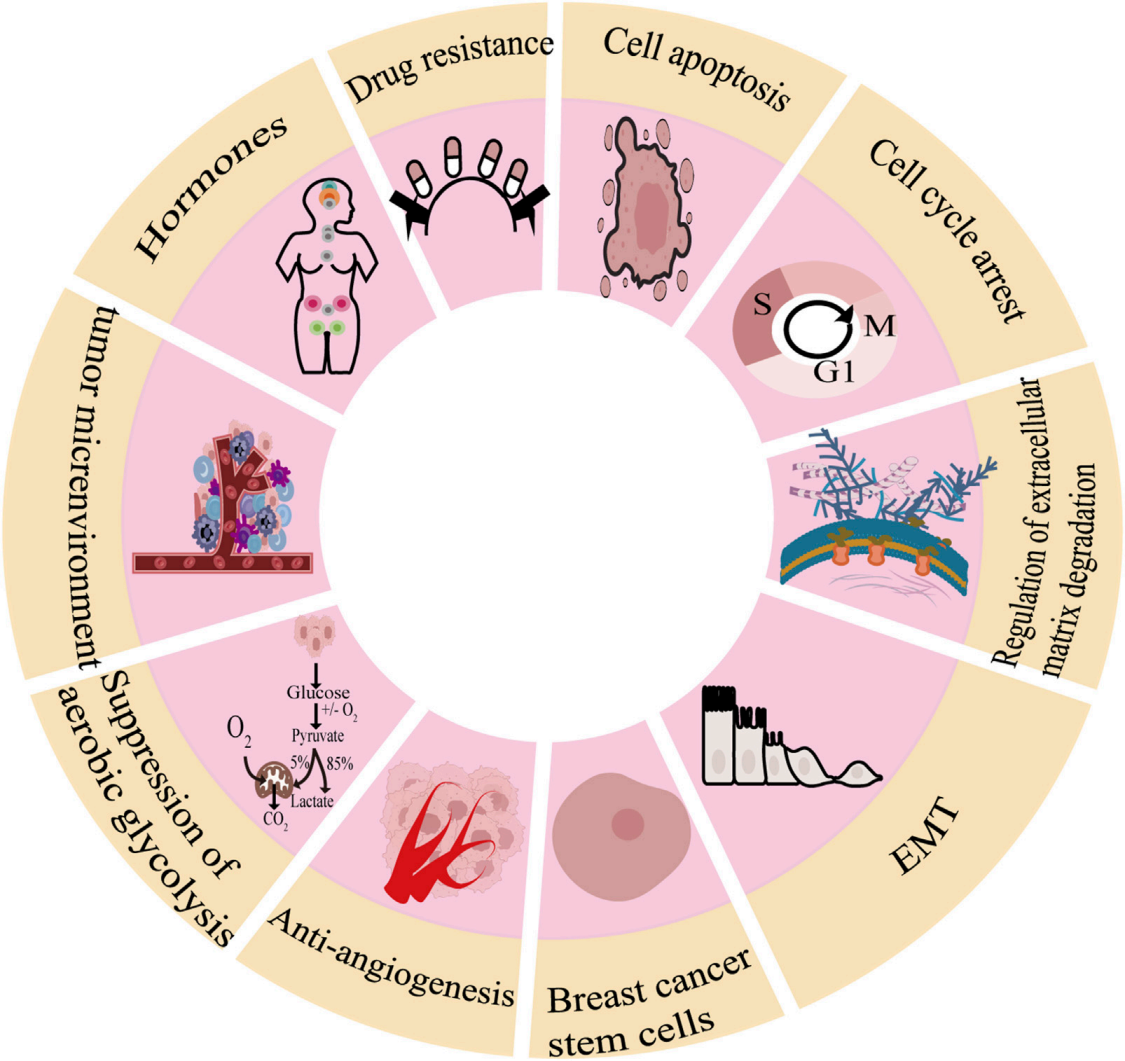
Molecular pathogenesis of breast cancer.

**FIGURE 2:**
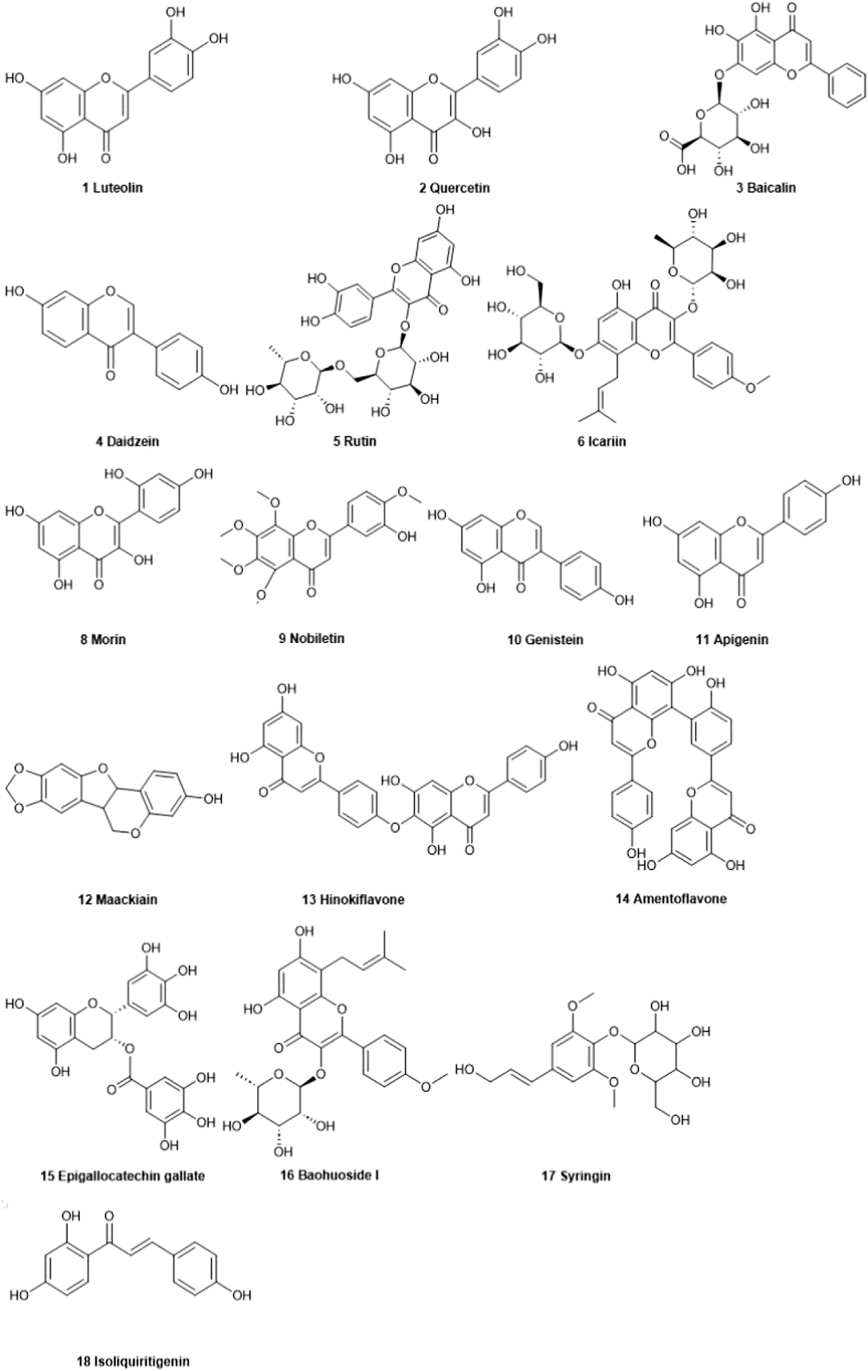
Chemical structure of flavonoids.

**FIGURE 3:**
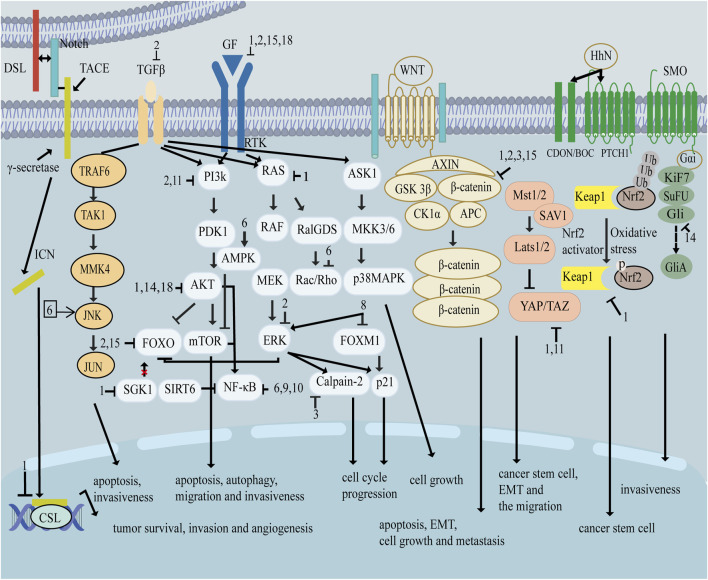
Signal pathway related to flavonoids inhibiting triple negative breast cancer.

As shown in Figure 1, there are many therapeutic targets that show potential clinical utility in the treatment of TNBC. The following will introduce the inhibitory effects of specific drugs on TNBC. Figure 2 shows chemical structure of flavonoids. Tables 1, 2 provide a concise overview of the pertinent research findings from both *in vivo* and *in vitro* trials on various medications. Figure 3 summarizes the relevant pathways.

**TABLE 1:** *In vitro* study on the inhibition of triple-negative breast cancer by flavonoids.

Bioactive molecules	Cell lines	Action on breast cancer	References
Luteolin	MDA-MB-231, 4T1	SGK1↓, p-FOXO3a↓, FOXO3a↑, BNIP3↑, Bcl-2↑, Bax↑, Bim↑, Beclin-1↑, LC3Ⅱ/Ⅰ↑, and P62↓	Wu et al. (2023)
	BT-20	phosphor-AKT↓, mTOR↓, N-cadherin↓, vimentin↓,E-cadherin↑, B-catenin↑, MMP-2↓, MMP-9↓, acetylated H3K27↓, and acetylated H3K56↓	Wu et al. (2021)
	MDA-MB-231, 4T1	CTGF↓, CYR2↓, p-YAP↑, p-TAZ↑, fibronectin↓, N-cadherin↓, vimentin↓, and E-cadherin↑	Cao et al. (2020)
	MDA-MB-231, BT5-49	vimentin↓, F-actin↓, E-cadherin↑, claudin↑, N-cadherin↓, Snail↓, Slug↓, and β-catenin↓	Lin et al. (2017)
	MDA-MB-231	ABCG2↓, Nanog↓, Oct4↓, CD44↓, Nrf2↓, HO-1↓, Sirt3↓, and Cripto-1↓	Tsai et al. (2021)
	MDA-MB-231	VEGF↓	Cook et al. (2016)
	LM2		
	MDA-MB-231	VEGF↓, Notch-1↓, Hes-1↓, Hey↓, Cyclin D1↓, and MMP↓	Sun et al. (2015)
	MDA-MB-453	Bax↑, Bcl-2↓, cleaved caspase-3↑, vimentin↑, Zeb1↑	Gao et al. (2019)
		N-cadherin↑, E-cadherin↓, Ras↓, Raf↓, p-MEK↓, and P-ERK↓	
Quercetin	MDA-MB-231	PI3K↓, Akt↓, mTOR↓, PTEN↑, Bax↑, caspase-3↑, Bcl-2↓	Jiang et al. (2024)
	MDA-MB-231	Foxo3a↑ and p-JNK/JNK↑	Nguyen et al. (2017)
	MDA-MB-231	FASN↓, β-catenin↓, and Bcl-2↓	Sultan et al. (2017)
	MDA-MB-157		
	MDA-MB-453	HuR↓, CD44↓, and β-catenin↓	Umar et al. (2022)
	MDA-MB-231	vimentin↓, E-cadherin↑, relative E-cadherin↑, β-catenin↑, cyclin D1↓, c-Myc↓, and p-AKT↓	Srinivasan et al. (2016)
	MDA-MB-231	MMP-2↓, MMP-9↓, VEGF↓, PKM2↓, GLUT1↓, LDHA↓, C3Ⅱ/LC3Ⅰ↑, p-AKT/AKT↓, p-mTOR/mTOR↓, and p-p70s6k/p70S6K	Jia et al. (2018)
	MDA-MB-231	miR-146a↑, bax↑, caspase-3↑, and EGFR↑	Tao et al. (2015)
	MDA-MB-231	P-gp↓, BCRP↓, and MRP1↓	Li et al. (2018a)
	MDA-MB-231	P-glycoprotein↓, BCRP↓, and MRP2↓	Louisa and Wardhani (2019)
	MDA-MB-231, MDA-MB-468	p-ERK1/2↓, c-Mye↓, PCNA↓, MMP2↓, and E-cadherin↑	Zhang et al. (2024b)
	4T1	P-JAK2/JAK2↓, P-STAT3/STAT3↓, the proportion of Treg cells↓, IL-10↓, TNF-α↑, Cleaved caspase-3/Caspase-3↑	Liao et al. (2024)
Baicalin	MDA-MB-231	MAPK↓, MMP-2↓, MMP-9↓, uPA↓, and uPAR↓	Wang et al. (2013a)
	MDA-MB-231, 4T1	E-cadherin↑, claudin↑, N-cadherin↓, vimentin↓, Snail↓, Slug↓, and β-catenin↓	Zhou et al. (2017)
	MDA-MB-231, osteoblasts	OPG↓, RANKL↑, Cytochrome C↑, caspase-3↑, caspase-8↑, caspase-9↑, p-mTOR↓, p-p70↓, OCN↓, and ALP↓	Wang et al. (2020a)
	MCF-7, MDA-MB-231	miR-338 3p↑ and MORC4↓	Duan et al. (2019)
	MDA-MB-231	TGF-β1↓, ZEB1↓, N-cadherin↓, E-cadherin↑, lncRNA-MALAT1↓, and miR-200c↑	Li et al. (2022)
Baicalein	MDA-MB-231	Bax↑, Bcl-2↓, LC3B↑, BECN1↑, IκB↑, p-AKT/AKT↓, and p-mTOR/mTOR↓	Yan et al. (2018)
	MDA-MB-231	SATB1↓, vimentin↓, Snail↓, E-cadherin↑, Wnt1↓, β-Catenin↓, Axin2↓, and Cyclin D1↓	Ma et al. (2016)
	MDA-MB-231	LC3B II/I↑, p62↓, PINK1↑, and parkin↑	Hua et al. (2023)
Daidzein	MDA-MB-453	CDK2↓, CDK4↓, CyclinD↓, CDK1↓, p27Cip2↓, and caspase-9↑	Choi and Kim (2008)
Rutin	MDA-MB-231	p-c-Met↓	Elsayed et al. (2017)
	MDA-MB-231	ASK1↑ and JNK↑	Suganya et al. (2022)
		MKI67↑, VIM↑, CDH2↑, FN1↑, VEGFA↑, CDH1↓, THBS1↓, N-cadherin↑, VEGFA↑, E-cadherin↓, and thrombospondin 1↓	Hajimehdipoor et al. (2023)
	4T1	miR-129-1-3p↑	Hajimehdipoor et al. (2023)
	MDA-MB-231	P-gp↓ and BCRP↓	Iriti et al. (2017)
Icariin	MDA-MB-468, 4T1	PCNA↓, LC3B-I↑, LC3B-II↑, BECN1↑, P62↓,	Zhao et al. (2024)
	MDA-MB-231	lncRNA NEAT1↓, E-cadherin↑, ZO-1↑, Vimentin↓, Smad2↓, P-Smad2↓, Smad2/3↓, TGFβ1↓, EpCAM↓, and SOX4↓	Song et al. (2024)
	MDA-MB-231, MDA-MB-453	ER-α36↓, EGFR↓, Bcl-2↓, BAX↑, cleaved PARP↑, cleaved caspase-3↑, p-MEK↓, and p-ERK↓	Wang et al. (2017)
	MDA-MB-436, Hs 578T	c-IAP2↓, p-c-Jun↓, p-JNK↓, and JNK↓	Gao et al. (2023)
	MDA-MB-231	Bcl-2↓, Bax↑, cleaved caspase-3↓, SIRT6↑, H3K9 Ac↓,	Song et al. (2020)
		E-cadherin↑, N-cadherin↓, and MMP-2↓	
Kaempferol	MDA-MB-231	Active RhoA↓, Active Rac1↓, vimentin↑, Snail↑, Slug↑, MMP2↓, and MMP9↓	Li et al. (2017)
	MDA-MB-231, *ex-vivo* grown primary breast tumor cells	CD44↓, OCT4↓, NANOG↓, and MDR1↓	Nandi et al. (2022)
Morin	MDA-MB-231	P-H2A.X↑, RAD51↓, survivin↓, Ku80↓, p-ERK↑, p21↑, cyclin A2↓, cyclin B1↓, p-ERK↑, and FOXM1↓	Maharjan et al. (2021)
Nobiletin	BT-549	JAZF1↓, NLRP3↑, cleaved caspase-1↑, ASC↑, IL-1β↑, IL-18↑, p-p65/t-p65↑, and p-*IkB/t-IkB↑*	Wang et al. (2021)
	MDA-MB-468	cyclin-D1↓, ERK1/2↓, p-AKT↓, p-mTOR↓, p21↑, and Bcl-xL↓	Wang et al. (2021)
Nobiletin	MDA-MB-231	IkBα ↑	Kim et al. (2022)
Genistein	MDA-MB-231	Notch-1↓, cyclin B1↓, Bcl-2↓, and Bcl-xL↓	Pan et al. (2012)
Apigenin	MDA-MB-231	IL-6↓, Snail↓, and N-cadherin↓	Lee et al. (2019b)
	MDA-MB-231	CD44/CD24+↓, YAP/TAZ↓, CTGF↓, and CYR61↓	Li et al. (2018b)
	MDA-MB-436		
	4T1	Bcl-2/Bax↓, caspase-3/cleaved-caspase-3, ROS↑, p-PI3K/PI3K↓, p-AKT/AKT↓, and Nrf2↓	Zhang et al. (2024a)
Maackiain	MDA-MB-231 BT549	N-cadherin↓, vimentin↓, E-cadherin↑, miR-374a↓, and GADD24α↑	Peng et al. (2022)
Amentoflavone	SUM159	CD44↑, ALDH1↑, CD24↓, Gli1↓, Smo↓, Sufu↑, Ptch1↑, NANOG↓, and OCT4↓	Bao et al. (2019)
ECCG	MDA-MB-231	β-catenin↓, p-Akt↓, and cyclin D1↓	Hong et al. (2017)
	MDA-MB-231	N-cadherin↓, Vimentin↓, ZO-1↑, occludin↑, and GOLM 1↓	Xie et al. (2021)
	Hs578T	caspase-1↑, caspase-3↑, caspase-4↑ and PYCARD↑	Hong et al. (2017)
	MDA-MB-231	SCUBE2↑, E-cadherin↑, vimentin↓, and total DNMTs activity↓	Sheng et al. (2019)
	MDA-MB-436	ER-α36↓, EGFR↓, p-ERK1/2↓, and p-AKT↓	Pan et al. (2016)
	NF639	E-cadherin↑, MTA3↑, γ-catenin↑, Snail↓, FOXO3a↑, ERα↑, VASP↓, and Rac1↓	Belguise et al. (2007)
Baohuoside I	BT549, 4T1	Vimentin↓ and MMP2↓	Wang et al. (2020b)
Syringin	MDA-MB-231	CDK4↓, p21↑, XIAP↓, cleaved caspase-9↑, and cleaved PARP↑	Lee et al. (2019a)
	MDA-MB-231	p-EGFR↓, p-HRAS↓, p-MAP2K1↓, p-ERK1/2↓, BAX↑, cleaved caspase-3↑, Bcl-2↓, p-PIK3CA↓, p-AKT↓, p-p65↓, and COX-2↓	Wang et al. (2022a)
Isoliquiritigenin	MDA-MB-231	Bcl-2↓, Bax↑, cleaved caspase-3↑, cleaved PARP↑, mTOR↓, p-mTOR↓, ULK1↑, p-ULK1↑, p62↑, Beclin1↑, and LC↑	Lin et al. (2020)
	MDA-MB-231	cytoplasmic Cyt C↑, cleaved caspase-9↑, miR-374a↓, PTEN↑, p-Akt↓, p-GSK3β↓, β-catenin↓	Peng et al. (2017)
	MDA-MB-231	VEGF↓ and HIF-1α↓	Wang et al. (2013b)

**TABLE 2:** *In vivo* study on the inhibition of triple-negative breast cancer by flavonoids.

Bioactive molecules	Study model	Daily experimental group dose	Daily control group drugs	Administration method	Action on breast cancer	References
Luteolin	4T1 mouse models	20 mg/kg, 40 mg/kg	10% DMSO	injection every 7 days for 3 weeks	SGK1↓, p-Foxo3a↓, FOXO3a↑, BNIP3↑, LC3↑, Bax↑, Bim↑, LC3-II/I↑, and Beclin-1↑	Wu et al. (2023)
	4T1 xenograft murine models	40 mg/kg	vehicle	injection daily for 18 days	YAP/TAZ↓	Cao et al. (2020)
	4T1 xenograft murine models	100 mg/kg	0 mg/kg	injection three times every week for 8 weeks	vimentin↓, Slug↓, and β-catenin↓	Lin et al. (2017)
	MDA-MB-231 (4,175) LM2 lung metastasis xenograft model	40 mg/kg	70% DMSO/30% PBS	injection daily for the first week and once every 2 days until 42 days	lung colonies↓	Cook et al. (2016)
	MDA-MB-231 mouse model	40 mg/kg, 20 mg/kg	PBS, Paclitaxel 20 mg/kg/d	tail vein injections daily for 14 days	breast cancer xenograft growth↓	Sun et al. (2015)
Quercetin	MDA-MB-231 mouse model	50 mg/kg	DMSO/0.9%physiologic saline (1:0.5)	injection daily for 25 days	FASN↓	Sultan et al. (2017)
	4T1 mouse model	50 mg/kg Que, 5 mg/kg Dox plus 50 mg/kg Cyc plus 50 mg/kg Que	0.9% saline, 5 mg/kg Dox plus 50 mg/kg Cyc	injection every other day for 6 administrations	AST↓, LDH↓, and CK↓	Eskiler et al. (2020)
	4T1 mouse model	50 mg/kg Que, physical constraint plus 50 mg/kg Que	0.9% saline, physical constraint	4 h of physical constraint per day for 12 days; injection every other day for 12 days	the average tumor volume↓, chronic stress-mediated lung metastasis of tumors↓	Zhang et al. (2024b)
	4T1 mouse model	quercetin 1% plus Cyclophosphamide; quercetin 2.5% plus Cyclophosphamide; and quercetin 5% plus Cyclophosphamide	vehicle, Cyclophosphamide 50 mg kg i.p.	cyclophosphamide injection on 7 days and 21 days, 2 injections in total	frequency of T cells and NK cells↑, frequency of Treg cells↓	Manni et al. (2023)
		quercetin 1%; quercetin 2.5%; quercetin 5%		culture for 27 days		
	4T1 mouse model	50 mg/kg, 100 mg/kg, 200 mg/kg	0.5% CMC-Na, 50 mg/kg of 5-FU, formulated in saline	intragastric administration for 14 days	PCNA↓	Liao et al. (2024)
Baicalin	4T1 mouse model	100 mg/kg	PBS	injection every 3 days until 6 weeks	vimentin↓ and Slug↓	Zhou et al. (2017)
Baicalein	MDA-MB-231 mouse model	100 mg/kg	1% carboxymethyl cellulose sodium	intra-gastric gavage daily for 21 days	p-AKT↓, Bax↑, and LC3↑	Yan et al. (2018)
Baicalein	MDA-MB-231 cancer cells mixed with M2 macrophages	50 mg/kg	vehicle	intra-gastric gavage every other day for 5 weeks	iNOS↑, E-cadherin↑, TGF-β1↓, CD206↓, N-cadherin↓, Vimentin↓, and p-Akt↓	Yan et al. (2018)
	MDA-MB-231 mouse model	50 mg/kg, 100 mg/kg	NS	intragastric gavage daily for 15 days	SATB1↓, Wnt1↓, β-catenin ↓, vimentin↓, SNAIL↓, and E-cadherin↑	Ma et al. (2016)
Icariin	4T1 mouse model	20 mg/kg, 40 mg/kg	control group drugs	injection daily for 15 days	p-mTOR↓, p-AMPK↑, and p-ULK1↑	Zhao et al. (2024)
Rutin	MDA-MB-231 xenograft models	30 mg/kg	vehicle	injection 3X/week/mice/i.p. for 28 days	breast cancer xenograft growth↓	Elsayed et al. (2017)
Nobiletin	MDA-MB-231 xenograft model	10 mg/kg	DMSO or DTX (10 mg/kg) inject once a week for 4 weeks	NOB-containing (0.1%) diet for 43 days	Ki67↓, TNF-α↓, and p-p65↓	Kim et al. (2022)
Genistein	MDA-MB-231 mouse model	25 mg/kg apigenin dissolved in 100 μL vehicle	100 μL vehicle (20% DMSO, 10% ethanol, 30% KolliphorEL, and 40% PBS)	injected daily for 28 days	ASI CFLAR_S_↓, MAP3K7-A↓, ASI BAX_L_↑, and CCNL2↑	Sudhakaran et al. (2023)
Apigenin	MDA-MB-231 mouse model	25 mg/kg, 50 mg/kg	drinking water	diet daily for 2 weeks	pSTAT3↓, pERK↓, PI3K↓, and pAkt↓	Lee et al. (2019b)
	HCI001 Patient-derived xenograft model	1, 5, 10, 25, 50 μM	DMSO	culture for 5 days	breast cancer xenograft growth↓ and viability↓	Sudhakaran et al. (2020)
	4T1 mouse model	25 mg/kg, 50 mg/kg, and 100 mg/kg	40% PEG400% and 60% double-distilled water, 2 mg/kg/week of DOX	injection for 21 days	PCNA↓ and proportion of Treg cells↓	Zhang et al. (2024a)
Hinokiflavone	MDA-MB-231 mouse model	20 mg/kg, 40 mg/kg	corn oil treated	injected daily for 3 weeks	Ki67↓ and MMP-2↓	Huang et al. (2020)
Baohuoside I	4T1-Luc xenograft mouse model	10 mg/kg, 20 mg/kg	0.5% carboxymethylcellulose sodium solution	injected daily for 36 days	vimentin↓, ALDH1A1↓, and β-catenin↓	Wang et al. (2020b)
		TAMs +20 mg/kg/d BHS group, TAMs + CXCL1 + 20 mg/kg/d BHS group	TAMs group	injected daily for 25 days		
Isoliquiritigenin	MDA-MB-231 mouse model	25 mg/kg/d, 50 mg/kg/d	PBS	injected daily for 25 days	MVD↓, VEGF↓, p-VEGFR-2↓, and MMP2↓	Wang et al. (2013b)
	MDA-MB-231 mouse model	2.5 mg/kg, 5.0 mg/kg	0.25 mL/mouse PBS	oral gavage once daily for 2 weeks and for an additional 25 days after implantation	Ki-67↑ and VEGF↓	Lin et al. (2020)

### 3.1 Luteolin

Luteolin is a flavonoid metabolite derived from the foliage and stems of mignonette plants. Additionally, it serves as a key metabolite of *Lonicera japonica* Thunb [Caprifoliaceae], *Perilla frutescens* (L.). Britton [Lamiaceae], and *Chrysanthemum indicum* L. [Asteraceae]. Luteolin possesses antioxidant, pro-oxidant, antibacterial, anti-inflammatory, and cancer-preventive properties (Rakoczy et al., 2023). The IC50 values of luteolin after 48 h of treatment were 39.31 μM for MDA-MB-231 cells and 63.06 μM for 4T1 cells.

Luteolin suppresses TNBC by inducing apoptosis and autophagy through the SGK1-FOXO3a-BNIP3 signaling pathway (Wu et al., 2023). AKT/mTOR-inducing H3K27Ac and H3K56Ac levels were decreased by luteolin, which in turn controlled MMP9 production, thereby suppressing the growth and metastasis of TNBC via the androgen receptors (Wu et al., 2021). Luteolin inhibits YAP/TAZ activity, suppressing EMT and the migration of TNBC (Cao et al., 2020), effectively inhibiting TNBC metastasis by reversing EMT through the suppression of β-catenin (Lin et al., 2017). Luteolin effectively reduced the characteristic features of breast cancer stemness by inhibiting the expressions regulated by Nrf2 (Tsai et al., 2021). Furthermore, luteolin inhibited the metastasis of MDA-MB-435 and MDA231-LM2-4175 cells to the lungs *in vivo*, and its capacity to prevent the generation of VEGF and block kinase domain receptor-mediated activity may contribute to its anti-metastatic effects (Cook et al., 2016). Luteolin effectively suppresses Notch signaling by modulating specific miRNAs involved in tumor growth. These include upregulated miR-34a, miR-139-5p, miR-181a, miR-224, and miR-246, and downregulated miR-155 (Sun et al., 2015). The anti-tumor effects of luteolin on TNBC cell growth and EMT may be attributed to the inhibition of Ras/Raf/MEK/ERK signaling, which is suppressed by miR-203 (Gao et al., 2019).

### 3.2 Quercetin

Quercetin is a flavonol metabolite obtained as a polyphenolic flavonoid found in various several Chinese herbal medicines, including *Oldenlandia diffusa* (Willd.) Roxb [Rubiaceae], *Lobelia chinensis* Lour. [Campanulaceae] *Glehnia littoralis* (A.Gray) F. Schmidt ex Miq. [Apiaceae], *Ginkgo biloba* L. [Ginkgoaceae], and others. Quercetin, a biologically active flavonoid, has demonstrated anti-diabetic, antioxidant, anti-cancer, and anti-aging characteristics (Wang G. et al., 2022). The IC50 values for MDA-MB-231 and MDA-MB-157 cells after 48 h of treatment were calculated to be 230 ± 3 μM and 415 ± 4 μM, respectively.

In TNBC, Quercetin suppresses breast cancer cell growth and survival by targeting the Akt/mTOR/PTEN signaling pathway (Jiang et al., 2024). Quercetin alters FOXO3a signaling and causes apoptosis and cell cycle arrest (Nguyen et al., 2017). By suppressing fatty acid synthase and β-catenin, quercetin induces apoptosis in TNBC cells (Sultan et al., 2017). Quercetin inhibits the progression and migration of TNBC cells by impairing HuR activity (Umar et al., 2022). Quercetin induces EMT by influencing the positioning of β-catenin within the cell nucleus and controlling the expression of genes targeted by β-catenin (Srinivasan et al., 2016). Quercetin controls the β-catenin signaling pathway and decreases the movement of TNBC cells. Quercetin hampers the growth of tumors, suppresses oncocytes proliferation, and induces tumor necrosis. Furthermore, it also inhibits cancer cell migration by suppressing glycolysis through the induction of autophagy mediated by the Akt-mTOR pathway (Jia et al., 2018). Quercetin inhibits the growth of human breast cancer cells by increasing miR-146a expression, followed by the induction of apoptosis via cystatinase-3 activation and the mitochondria-dependent pathway and inhibition of invasion via downregulation of epidermal growth factor receptor (EGFR) expression (Tao et al., 2015). Quercetin could inhibit chronic stress-induced ERK1/2 activity in TNBC cells, thereby weakening the potential for TNBC growth and metastasis (Zhang J. et al., 2024). Quercetin boosts the efficiency of doxorubicin in treating human breast cancer cells while minimizing its harmful side effects. This is achieved by decreasing the expression of efflux ABC transporters (Li S. et al., 2018). The efficacy of sorafenib is improved by quercetin, which reduces the levels of two drug efflux transporters: P-glycoprotein and BCRP (Louisa and Wardhani, 2019). Quercetin reduces the harmful effects of doxorubicin-cyclophosphamide treatment on the heart, enhancing its ability to treat TNBC. Quercetin may reduce the harmful effects of AC-induced heart damage by preventing the buildup of ROS and stimulating the ERK1/2 pathway in heart muscle cells. Thus, quercetin may augment the anti-cancer efficacy of AC by suppressing ROS accumulation and inhibiting the ERK1/2 pathway in TNBC cells (Zhang et al., 2022). Quercetin reverses talazopanib resistance in BRCA1-mutated TNBC cells by improving cytotoxicity and apoptosis (Eskiler et al., 2020). By modulating the IL-6/JAK2/STAT3 signaling pathway, quercetin reduces the number of Treg cells and activates the anti-tumor immune response (Liao et al., 2024). Cyclophosphamide and quercetin increased the overall occurrence of T cells and NK cells in the body, while decreasing the occurrence of Treg cells, which are associated with suppressing tumour growth (Manni et al., 2023).

### 3.3 Baicalin

Baicalin is the primary metabolite of *Scutellaria baicalensis* Georgi [Lamiaceae], *Oroxylum indicum* (L.) Kurz [Bignoniaceae], and *Pinellia ternata* (Thunb.) Makino [Araceae] has diverse effects, including anti-cancer, anti-inflammatory, anti-apoptotic, and antibacterial properties (Bajek-Bil et al., 2023). The IC50 values of baicalein in MDA-MB-231 cells were 60.12 μM at 24 h, 27.98 μM at 48 h, and 19.01 μM at 72 h. Baicalin suppresses tumor growth in MDA-MB-231 cells by decreasing the expression of MMP-2, MMP-9, uPAR, and uPA by disrupting the p38MAPK signaling pathway (Wang X.-F. et al., 2013). Furthermore, baicalin targets β-catenin signaling to reverse EMT, thereby inhibiting the metastasis of breast cancer cells (Zhou et al., 2017). Baicalin substantially inhibited the proliferation of bone metastases, reduced bone degradation, diminished osteoclastogenesis of osteoclast progenitors, and inhibited the growth of metastasized MDA-MB-231 cells, thus maintaining bone mass (Wang B. et al., 2020). Baicalin reduces the viability, motility, and invasion of breast cancer cells by modulating MORC4 and miR-338-3p (Wang B. et al., 2020). Baicalin notably reduces breast cancer cell survivability, mobility, and invasiveness by controlling the TGF-β/lncRNA-MALAT1/miR-200c pathway (Li et al., 2022). Furthermore, Baicalein triggered programmed cell death and self-degradation in triple-negative breast cancer cells by blocking the PI3K/AKT pathway (Yan et al., 2018). Baicalein suppressed fibronectin-induced EMT by reducing calpain-2 activation and upregulation (Chen et al., 2019). Baicalein inhibited EMT in breast cancer by regulating the polarization of TAMs (Zhao et al., 2018). Baicalein may inhibit EMT, which is linked to the downregulation of the Wnt/β-catenin pathway and SATB1 to decrease breast cancer metastasis (Ma et al., 2016). By blocking the G-protein-coupled receptor 30 pathway, baicalein prevents 17-β-estradiol from causing BC cells to migrate, adhere, or invade (Shang et al., 2015). Baicalein decreased CDK1 activity through autophagy, increasing MDA-MB-231 cells’ susceptibility to doxorubicin (Hua et al., 2023).

### 3.4 Daidzein

Daidzein, an isoflavone, possesses significant nutritional value and is primarily derived from soy plants *Cyathula officinalis* K.C.Kuan [Amaranthaceae] and *Corethrodendron multijugum* (Maxim.) B.H.Choi and H.Ohashi [Fabaceae], and *Spatholobus suberectus* Dunn [Fabaceae]. Daidzein has biphasic effects on breast cancer cell proliferation and ERα expression, with either stimulatory or inhibitory effects. It exhibits pharmacological and therapeutic characteristics, including cholesterol-lowering, cardiovascular function improvement, anti-tumor, anti-fibrotic, and anti-diabetic properties (Ubaid et al., 2023). Daidzein’s anti-cancer properties in TNBC involve inducing cell cycle arrest, particularly at the G1 and G2/M phases (Choi and Kim, 2008).

### 3.5 Rutin

Rutin is a flavonoid that is derived from many plants, including *Artemisia argyi* H.Lév. and Vaniot [Asteraceae], *Podophyllum versipelle* Hance [Berberidaceae], *Ginkgo biloba* L. [Ginkgoaceae], and others. Rutin possesses a wide range of therapeutic properties, such as anti-allergic, antioxidant, anti-inflammatory, anti-cancer, and anti-diabetic effects (Yong et al., 2020). For a 24-h period, the rutin IC50 values for MDA-MB-231 cells were 40 ± 1.0 µM. Rutin acts as a c-Met inhibitor that inhibits TNBC cell proliferation, migration, and invasion (Elsayed et al., 2017), and its therapy in TNBC induces ER stress (Suganya et al., 2022). Rutin enhances the growth, migration, and pro-angiogenic functions of TNBC cell lines (Hajimehdipoor et al., 2023). Rutin can enhance the upregulation of miR-129-1-3p in 4T1 cells. By regulating the expression of GRIN2D, Calm1, and CaMKIIδ, miR-129-1-3p reduces calcium overcharge and downstream Ca^2+^ signaling in 4T1 cells, thereby contributing to the inhibition of breast tumor cell proliferation and metastasis (Li et al., 2021). Rutin and doxorubicin inhibit cell proliferation by arresting the cell cycle at the G2/M phase and inducing apoptosis through ER stress in MDA-MB-231 cells (Suganya et al., 2022). Rutin efficiently arrests the cell cycle in chemoresistant TNBC cells by inhibiting P-gp and BCRP pumps, reversing multidrug resistance, and restoring sensitivity to cyclophosphamide (Iriti et al., 2017).

### 3.6 Icariin

Icariin, a flavonoid extracted from *Epimedium sagittatum* (Siebold and Zucc.) Maxim. [Berberidaceae] has various beneficial properties, including anti-inflammatory, antioxidant, antidepressant, and aphrodisiac effects (Liu et al., 2023). Icariin triggers autophagy to hinder the progression of TNBC by stimulating the AMPK/mTOR/ULK1 signaling pathway (Zhao et al., 2024). By altering the lncRNA NEAT1/TGFβ/SMAD2 Signaling Pathway, Icariin regulates EMT and stem cell-like characteristics in breast cancer (Song et al., 2024). Icariin triggered cellular apoptosis and broke the positive regulatory loop between ER-α36 and EGFR in TNBC cells, resulting in a reduction of cell growth promoted by E2 in TNBC (Wang et al., 2017). It induces apoptosis by increasing ROS levels and suppressing the invasion of TNBC cells through the JNK/c-Jun signaling channel (Gao et al., 2023). Icariin induces apoptosis and inhibits migration of TNBC through the SIRT6/NF-κB signaling pathway. Icariin exhibits tumor growth inhibition and anti-lung metastasis effects in tumor animal models of MDA-MB-231 and 4T1 cells via the immunosuppressive microenvironment of the tumor (Song et al., 2020).

### 3.7 Kaempferol

Kaempferol is found in high levels in tea, *Paeonia lactiflora* Pall [Paeoniaceae], *Platycladus orientalis* (L.) Franco [Cupressaceae] and several other sources. It possesses notable antibacterial, antifungal, anti-cancer, antioxidant, antiprotozoal, and anti-inflammatory properties (Periferakis et al., 2022). Low-dose kaempferol inhibited TNBC cell migration and encroachment by plugging the RhoA and Rac1 signaling channels, whereas HER2 overexpression rescued both cell migration and RhoA and Rac1 activation in kaempferol-treated MDA-MB-231 cells (Li et al., 2017). The combined anticancer effect of kaempferol and verapamil is strengthened by deregulation the CD44-NANOG-MDR1-associated chemoresistance pathway in breast cancer stem cells (Nandi et al., 2022).

### 3.8 Morin

Morin, a widely recognized flavonoid derived from plants in the Moraceae family and the leaves of *Maclura tricuspidata* Carrière [Moraceae], has anti-inflammatory, anti-oxidant, anti-diabetic, anti-tumor, anti-hypertensive, antibacterial, and neuroprotective properties (Maharjan et al., 2021). MDA-MB-231 cell death triggered by morin results from prolonged interruption of the cell cycle, which occurs due to the activation of ERK and suppression of FOXM1 signaling pathways, leading to the stimulation of p21 expression. Morin suppresses FOXM1 and attenuates EGFR/STAT3 signaling pathways to sensitize TNBC cells to doxorubicin cytotoxicity (Maharjan et al., 2023).

### 3.9 Nobiletin

Nobiletin, a flavonoid extracted from *Citrus reticulata* Blanco [Rutaceae], exhibits many positive effects, including neuroprotection, cardiovascular protection, anti-metabolic disorder prevention, anti-cancer, anti-inflammatory, and antioxidant properties (Arshad et al., 2024). Nobiletin induces apoptosis and pyroptosis of TNBC cells via miR-200b/JAZF1/NF-κB axis (Wang et al., 2021). Nobiletin exhibits anti-cancer effects in TNBC cells via inducing apoptosis through Bcl-xL and causing cell cycle arrest in the G0/G1 phase (Chen et al., 2014). The anti-cancer effectiveness was improved by activating retinoic acid receptor-related orphan receptors with nobiletin. This enhancement is achieved by suppressing the IκB/NF-κB signaling pathway in TNBC. The concurrent use of nobiletin with either docetaxel or carboplatin effectively suppresses the proliferation of TNBC cells (Kim et al., 2022).

### 3.10 Genistein

Genistein is a prevalent isoflavone present in soy products. Genistein demonstrates anti-inflammatory, antioxidant, antibacterial, and antiviral properties. It impacts angiogenesis and estrogen synthesis and has pharmacological effects on diabetes and lipid metabolism (Sharifi-Rad et al., 2021). Eisenstein effectively attenuated complications in TNBC lacking ERs across all doses (Malik et al., 2023). Genistein suppresses the growth of TNBC cells by reducing the activity of NF-κB through the Notch-1 pathway (Pan et al., 2012). Genistein can effectively suppress the growth of TNBC cells by intricately modulating the cell cycle and the response to DNA damage (Fang et al., 2016). Genistein can potentially prevent and reverse AHR-dependent BRCA1 hypermethylation and restore ERα-mediated responsiveness. This makes TNBC more sensitive to estrogen therapy (Donovan et al., 2019).

### 3.11 Apigenin

Apigenin is found in several medicinal plants, such as *Plantago indica* L. [Plantaginaceae] and *Lobelia chinensis* Lour. [Campanulaceae], and *Mentha canadensis* L. [Lamiaceae]. Apigenin exhibits anti-tumor, cardioprotective, neuroprotective, and anti-inflammatory effects (Salehi et al., 2019). The IC50 values of apigenin in MDA-MB-231 and MDA-MB-436 cells were around 33 and 30 μM, respectively, after 72 h. Apigenin alters transcriptome-wide TNBC-specific alternative splicing, specifically in TNBC, leading to apoptosis and tumor growth inhibition (Sudhakaran et al., 2023). Apigenin inhibited the invasion of xenograft tumors derived from MDA-MB-231 by decreasing the IL-6-associated downstream signaling cascade (Lee H. H. et al., 2019). Apigenin inhibits the activation of the PI3K/Akt channels and the activity of integrin β4, resulting in a decrease in the metastasis of tumor cells to the lungs in nude mice and the occurrence of spontaneous intravasation and organ metastasis in chick embryos (Lee et al., 2008). Apigenin inhibits YAP/TAZ activity in TNBC cells and suppresses the stem cell-like properties of apigenin in TNBC cells, which is partially mediated by disturbing the YAP/TAZ-TEAD protein-protein interaction (Li Y.-W. et al., 2018). By concentrating on hnRNPA2, apigenin modulated the activity of ABCC4 and ABCG2 drug expulsion transporters and increased the sensitivity of TNBC spheroids to DOX-induced apoptosis (Sudhakaran et al., 2020). Apigenin boosts the suppressive impact of cisplatin on telomerase activity in TNBC cells (A.Aziz et al., 2017). Apigenin may induce apoptosis in breast cancer cells by activating the PI3K/AKT/Nrf2 pathway. Additionally, it can enhance the tumor immune microenvironment in mice with breast tumors, leading to the suppression of breast cancer growth (Zhang C. et al., 2024).

### 3.12 Maackiain

Maackiain is a flavonoid with several functions and is extracted from many Chinese herbs, including *Spatholobus suberectus* Dunn [Fabaceae] and *Sophora flavescens* Aiton [Fabaceae]. Maackiain exhibits many pharmacologic activities, including anti-cancer, anti-allergic, and anti-inflammatory effects (Huh et al., 2020). Maackiain regulates the miR-374a/GADD45A axis to suppress the beginning and marching of TNBC (Peng et al., 2022).

### 3.13 Hinokiflavone

Hinokiflavone is natural metabolites in several plants, such as *Platycladus orientalis* (L.) Franco [Cupressaceae], *Selaginella moellendorffii* Hieron [Selaginellaceae], and *Toxicodendron succedaneum* (L.). Kuntze [Anacardiaceae] exhibits various pharmacological effects, including anti-inflammatory, antiprotozoal, antioxidant, and anti-cancer effects (Goossens et al., 2021). Hinokiflavone inhibits the transformation of TNBC cells and leads to apoptosis via the EMT signaling pathway (Huang et al., 2020).

### 3.14 Amentoflavone

Amentoflavone is a natural biflavonoid found in several plants, such as *Ginkgo biloba* L. [Ginkgoaceae] and *Platycladus orientalis* (L.) Franco [Cupressaceae]. It exhibits anti-inflammatory, musculoskeletal protection, anti-microorganism, anti-oxidation, metabolic regulation, neuroprotection, radioprotection, antidepressant, and anti-carcinogenic effects (Xiong et al., 2021). Amentoflavone inhibits TNFα-induced Gli1 activation in breast tumor cells, resulting in reduced invasiveness of human breast cancer cells by interceding AKT/mTOR/S6K1 signaling and blocking the migration and invasiveness of MDA-MB-231 cells (Qiu et al., 2021). Amentoflavone suppresses the development of tumorspheres in SUM159 BCSCs by regulating the Hedgehog/Gli1 signaling pathway (Bao et al., 2019).

### 3.15 Epigallocatechin-3-gallate

Epigallocatechin-3-gallate (EGCG) is a polyphenolic metabolite extracted from traditional Chinese medicines such as *Ginkgo biloba* L. [Ginkgoaceae], *Eriobotrya japonica* (Thunb.) Lindl. [Rosaceae], and *Phyllanthus emblica* L. [Phyllanthaceae]. EGCG shows anti-fibrotic, anti-inflammatory, pro-apoptotic, and anti-tumorous effects (Mokra et al., 2022). EGCG suppresses the proliferation of MDA-MB-231 breast cancer cells by deactivating the β-catenin signaling system (Hong et al., 2017). By blocking VEGF expression, EGCG prevents human breast cancer cells from proliferating, migrating, and invading (Braicu et al., 2013). EGCG suppresses the migration of MDA-MB-231 cells by inhibiting GOLM1 via the HGF/HGFR/AKT/GSK-3β/β-catenin/c-Myc signaling pathway (Xie et al., 2021).EGCG induces cell death and apoptosis in TNBC Hs578 by activating the anti-apoptotic genes BAG3, XIAP, and RIPK2(Braicu et al., 2013). EGCG reduced SCUBE2 methylation status by reducing the expression and activity of DNA methyltransferase, thereby inhibiting cell migration and invasion (Sheng et al., 2019). By downregulating the positive feedback loop between ER-α36 and EGFR, EGCG effectively blocks the growth of both ER-negative stem cells and ER-negative progenitor cells. EGCG also stimulates FOXO3a, which in turn activates ERα and reverses the invasiveness of breast cancer cells. Furthermore, EGCG treatment induces the expression of constitutively active FOXO3a, which counteracts the transforming growth factor-β1-induced invasive phenotype and promotes an epithelial phenotype that depends on ERα expression and signaling (Belguise et al., 2007; Zhang et al., 2009). ECCG-induced growth suppression of ER-negative breast cancer stem and progenitor cells mediated by ERa36 (Pan et al., 2016).

### 3.16 Baohuoside I

Baohuoside I (BHS) is a flavonoid derived from the plant *Epimedium sagittatum* (Siebold and Zucc.) Maxim. [Berberidaceae] and has several pharmacological activities, including anti-osteoporotic and anti-tumor effects, enhancement of cognitive function, protection against cerebral ischemia-reperfusion injury, and neuroprotection (Agrawal et al., 2024). BHS effectively inhibits breast cancer metastasis and the activation of TAMs/CXCL1 in mouse breast cancer xenografts as well as in a zebrafish model of breast cancer xenotransplantation (Wang S. et al., 2020).

### 3.17 Syringoside

Syringoside is the primary active metabolite in *Eleutherococcus senticosus* (Rupr. and Maxim.) Maxim. [Araliaceae]. Syringoside has also been extracted from *Isatis tinctoria* L. [Brassicaceae], *Glehnia littoralis* (A.Gray) F.Schmidt ex Miq. [Apiaceae], and *Cirsium japonicum* DC. [Asteraceae]. It is primarily used as an anti-inflammatory agent. Syringin induces oxidative stress and inhibits TNBC growth (Lee C.-H. et al., 2019). Syringin inhibits the growth and migratory capabilities of TNBC cells while inducing apoptosis via the PI3K-AKT-PTGS2 and EGFR-RAS-RAF-MEK-ERK signaling pathways (Wang F. et al., 2022).

### 3.18 Isoliquiritigenin

Isoliquiritigenin, a flavonoid obtained from the plant *Glycyrrhiza glabra* L. [Fabaceae], has many pharmacological properties such as anticancer, antiaging, antioxidative, anti-inflammatory, and anti-diabetic effects (Zhao et al., 2019). Isoliquiritigenin efficiently suppress the growth of TNBC cells by inducing apoptotic cell death and promoting the accumulation of p62, which in turn stimulated autophagy-mediated apoptosis (Lin et al., 2020). Isoliquiritigenin suppresses the formation of new blood vessels in TNBC by targeting the VEGF/VEGFR-2 signaling pathway (Wang Z. et al., 2013). Isoliquiritigenin has the ability to regulate the miR-374a/PTEN/Akt pathway, which leads to the inhibition of breast cancer tumor growth and spread (Peng et al., 2017). Isoliquiritigenin derivative also had a more significant inhibitory effect on breast cancer cell viability, especially on MDA-MB-231 (Peng et al., 2020).

## 4 Conclusion

Treating TNBC is challenging because it has few effective therapeutic options. Recent studies have shown the anti-tumor properties of several natural metabolites, including flavonoids. There are many types of flavonoids, and how to screen and summarize the most effective ingredients from a large number of compounds remains a challenge. In addition, although the research on flavonoids has made certain progress, there is still a lack of a mature and unified framework to guide its application in clinical treatment. In this case, the concept of holism and treatment based on pattern differentiation of traditional Chinese medicine provide a new direction to explore the potential of flavonoids in tumor treatment. Based on the investigation of literature and ancient books, starting from ethnic folk medicines, clinical prescriptions and experience prescriptions, the selection is made with reference to the medicinal properties and efficacy of the drugs. The medicinal properties represented by the four qi and five flavors, meridians, ascending and descending, floating and sinking, etc., are theories for studying the properties and application rules of traditional Chinese medicine. Linking the efficacy of drugs with modern pharmacological mechanisms is the focus of future research. Various traditional Chinese medicines with functions such as nourishing the body and eliminating evil, clearing heat and detoxifying, promoting blood circulation and removing blood stasis have been proven to have direct or indirect anti-tumor effects.

This review provides a thorough overview of the various mechanisms by which flavonoids can effectively treat TNBC, including regulating cell proliferation and cell cycle, inhibiting cell invasion and metastasis, overcoming drug resistance, inducing apoptosis and autophagy, and inhibiting angiogenesis. Additionally, this review delves into the specific effects of different flavonoid metabolites or treating TNBC. Flavonoids are natural substances with anti-cancer activities. When combined with TCM, they can improve their efficacy and treatment outcomes. However, the bioavailability of flavonoids is low, their dosage is difficult to control, and are also potentially toxic. It is challenging to find definitive and universally applicable mechanisms of action in mechanism research. The structure-activity relationship should be conducted to design and optimize compounds. Clinical and basic research need to be closely integrated and verified to ensure clinical safety and effectiveness. The anti-cancer properties of flavonoids have opened new avenues for pharmacological intervention in TNBC.
